# *Mycobacterium tuberculosis* Peptides Presented by HLA-E Molecules Are Targets for Human CD8^+^ T-Cells with Cytotoxic as well as Regulatory Activity

**DOI:** 10.1371/journal.ppat.1000782

**Published:** 2010-02-26

**Authors:** Simone A. Joosten, Krista E. van Meijgaarden, Pascale C. van Weeren, Fatima Kazi, Annemieke Geluk, Nigel D. L. Savage, Jan W. Drijfhout, Darren R. Flower, Willem A. Hanekom, Michèl R. Klein, Tom H. M. Ottenhoff

**Affiliations:** 1 Department of Infectious Diseases, Leiden University Medical Center, Leiden, The Netherlands; 2 Department of Immunohematology and Blood Transfusion, Leiden University Medical Center, Leiden, The Netherlands; 3 The Jenner Institute, University of Oxford, Oxford, United Kingdom; 4 South African Tuberculosis Vaccine Initiative, School of Child and Adolescent Health, University of Cape Town, Cape Town, South Africa; Johns Hopkins School of Medicine, United States of America

## Abstract

Tuberculosis (TB) is an escalating global health problem and improved vaccines against TB are urgently needed. HLA-E restricted responses may be of interest for vaccine development since HLA-E displays very limited polymorphism (only 2 coding variants exist), and is not down-regulated by HIV-infection. The peptides from *Mycobacterium tuberculosis* (Mtb) potentially presented by HLA-E molecules, however, are unknown. Here we describe human T-cell responses to Mtb-derived peptides containing predicted HLA-E binding motifs and binding-affinity for HLA-E. We observed CD8^+^ T-cell proliferation to the majority of the 69 peptides tested in Mtb responsive adults as well as in BCG-vaccinated infants. CD8^+^ T-cells were cytotoxic against target-cells transfected with HLA-E only in the presence of specific peptide. These T cells were also able to lyse *M. bovis* BCG infected, but not control monocytes, suggesting recognition of antigens during mycobacterial infection. In addition, peptide induced CD8^+^ T-cells also displayed regulatory activity, since they inhibited T-cell proliferation. This regulatory activity was cell contact-dependent, and at least partly dependent on membrane-bound TGF-β. Our results significantly increase our understanding of the human immune response to Mtb by identification of CD8^+^ T-cell responses to novel HLA-E binding peptides of Mtb, which have cytotoxic as well as immunoregulatory activity.

## Author Summary

*Mycobacterium tuberculosis* (Mtb) has infected about one-third of the world population, resulting in fatal pulmonary tuberculosis (TB) in 1.5 million people annually. Vaccination against Mtb has decreased disease incidence in young children but does not prevent pulmonary TB in adults. The immune response against Mtb comprises multiple players, one of which is the CD8^+^ T-cell. CD8^+^ T-cells recognize infected cells because Mtb derived peptides are presented on HLA class I molecules. Here, we studied the non-classical HLA molecule HLA-E as presenter of Mtb antigens. The Mtb genome contained multiple sequences that can be presented by human HLA-E. These peptides were recognized by CD8^+^ T-cells from healthy individuals that were sensitized to Mtb, resulting in CD8^+^ T-cell proliferation. These T-cells lysed mycobacterium infected cells in a HLA-E restricted manner. Additionally, these T-cells also inhibited proliferation of other T-cells in their vicinity, a property of regulatory T-cells. The dual functionality of these T-cells makes them interesting players during infection, probably balancing their function depending on the environment. These findings contribute to our understanding of the immune response towards Mtb and will be helpful in designing new and improved vaccines against TB.

## Introduction

Tuberculosis (TB) remains a major health burden with one-third of the world population infected with *Mycobacterium tuberculosis* (Mtb) and 1.7 million deaths annually [1]. The currently available vaccine, *M. bovis Bacillus Calmette Guerin* (BCG), induces variable protection; although it protects well against severe childhood forms of TB, its ability to protect against pulmonary TB regardless of age is highly variable and at best partly [2],[3] urging the search for better vaccines [4]. The HIV pandemic has increased TB disease incidence in recent years, and vaccines which are effective and safe in HIV-infected individuals are urgently needed. After decades of neglect, the search for new vaccines against TB has only recently been reinitiated, but thus far few if any vaccines have been able to induce protection superior to that of BCG in animal models [4].

Optimal vaccine development requires insight into the mechanisms of protective host immunity. The immune response elicited upon Mtb infection is complex and incompletely understood. One of the most studied effector molecules of anti-Mtb immunity is IFNγ, which is produced predominantly by CD4^+^ T- and NK-cells. Effector immunity mediated by CD8^+^ T-cells has been less well characterized, and work reported thus far has focused almost exclusively on CD8^+^ T-cell responses restricted by classical HLA class Ia or CD1 a,b,c molecules [5]. The human HLA class I family comprises both classical (class Ia) and non-classical (class Ib) members. The former (i.e. HLA-A,-B,-C) molecules are highly polymorphic and comprise of 506, 872 and 274 different protein variants respectively, with large variations in potential bound peptides. HLA class Ib genes, instead, display limited polymorphism: there are 3, 4 and 10 protein variants described for HLA-E, -F and -G, respectively [6]. This minor variation between class Ib alleles is remarkable and suggests an evolutionary distinct role for class Ia and class Ib molecules. Non-classical HLA class I molecules including HLA-E are able to function as antigen presenting molecules for CD8^+^ T-cells, and can present both self and foreign (pathogen derived) antigens [7]–[9]. HLA-E presented self antigens include the signal sequences of classical HLA class I molecules and sequences from TCR-Vβ chains, the latter of which are recognized by CD8^+^ T-cells with cytotoxic (CTL) activity [10]. There is only one single amino acid difference between the different HLA-E proteins; this coding variation is located at position 107 (arginine to glycine) on the loop between β-strands in the α2 domain of the heavy chain, outside the peptide binding groove [11]. The lack of allelic variation in the peptide binding groove limits the number of possible peptides that can bind to HLA-E, and it is highly likely that similar peptides can be presented by both HLA-E variants [11]. The frequency of both variants, called HLA-E^R^ (E*0101) and HLA-E^G^ (E*0103) is equal amongst different populations suggesting balanced selection in divers populations [12]. Whether HLA-E^R^ and HLA-E^G^ can display functional differences has not been studied in detail [11],[12], but it has been demonstrated that HLA-E^G^ homozygous cells express higher levels of HLA-E and had higher peptide binding affinity, although this was tested in rather artificial models only [11].

Recognition of peptides presented by HLA-E may result in CD8^+^ effector T-cell activation [7]–[9]. For example, HLA-E can present antigens derived from pathogens including Mtb [13], *cytomegalovirus* [7] and *Salmonella typhi* [9]. The nature of the Mtb antigen(s) recognized by the only two reported human HLA-E restricted CD8^+^ T-cell clones, which produced IFNγ after co-incubation with Mtb-infected dendritic cells, remains unknown [13].

In addition to its limited genetic polymorphism, HLA-E offers another potential advantage in relation to vaccination in the context of HIV, a highly prevalent co-infection in TB. In Southern Africa alone approximately 70% of TB-patients are also HIV-infected [14]. HIV infection down-regulates expression of HLA-A and -B molecules through its Nef proteins, thus decreasing antigen presentation capacities [15]. In contrast, HLA-E is resistant to HIV-nef-mediated down-regulation due to a single amino acid substitution in the HLA-E cytoplasmic tail [16]. Persistent expression of HLA-E during HIV infection renders the HIV infected cells resistant to NK-mediated lysis [16]. Thus, while HIV might affect antigen presenting cells of the myeloid lineage like monocytes and macrophages [17] and inhibit antigen presentation by class Ia molecules, HLA-E dependent antigen presentation is likely to be less affected by HIV co-infection. Thus, targeting Mtb specific HLA-E restricted immunity by vaccination may be a novel and advantageous approach for several reasons. Furthermore, if BCG vaccination would already be able to prime HLA-E restricted T-cell responses, HLA-E peptide based vaccines might be able to boost BCG induced responses.

To date, most studies on HLA-E have focused on NK cells, which can recognize and kill target cells via cognate HLA-E/CD94-NKG2A interactions. As described above, a limited number of studies has shown that direct recognition of pathogen-derived antigen presented by HLA-E can occur via the T-cell receptor (TCR) [18],[19]. Recognition of specific peptides presented by HLA-E was found to result in CTL activity directed towards the peptide presenting cell [18],[19]. The molecular interactions between the TCR and HLA-E have not been studied extensively, and particularly, HLA-E peptide binding motifs have been determined only in relation to interactions of HLA-E with CD94/NKG2 receptors [20]. The peptide anchor residues critical for peptide binding to HLA-E might be conserved in both cases, but the residues at position 5 and 8, which are critically involved in the interaction with NKG2 [20] may be more variable for interactions with the TCR. However, in a study analyzing recognition of CMV UL40 derived peptide by a single TCR, peptide position 8 seemed critical for discrimination between self and non-self [21]. Crystallography revealed an interaction that mimicked the typical TCR-MHC class Ia complexes [21]. T-cells restricted to the mouse equivalent of HLA-E, Qa-1, are positively selected in the thymus, demonstrating specific recognition of Qa-1-peptide complexes in early T-cell development [22]. Thymic selection of Qa-1 restricted T-cells furthermore suggests positive selection of such T-cells *in vivo* as relevant immune players. Interestingly, detailed analysis of Qa-1 restricted CD8 T-cells in mice revealed suppressive capacities of these cells [23]. Suppression was specifically directed towards T-cells with intermediate avidity independent of the antigen, thus downregulating both self and non-self specific T-cells [24].

To start unraveling antigen specific human HLA-E restricted T-cell responses in the context of TB, we have used bioinformatics, HLA-E peptide-binding assays and functional characterization of the responding T-cells, in order to identify and characterize potential CD8^+^ T-cells that recognize Mtb peptides in the context of HLA-E.

## Results

### Epitope prediction

In terms of its bound peptidome, HLA-E remains a largely uncharacterized MHC class I molecule. This prompted us to use bioinformatics and *in vitro* peptide/HLA-E binding affinity assays to identify possible T-cell epitopes presented by this molecule.

Given the limitation of available resources, a highly guided approach was used which combined the use of legacy peptides, including known HLA-E binders (i.e. MHC class I leader sequences), the use of partial binding data [20] and iterated use of discriminant analysis (DA) and diversity analysis to suggest peptides for testing (Table 1) [25].

**Table 1:** Summary of peptide characteristics.

#	Sequence	Derived from	Accession number*	HLA-E motif score^a^	Discrimination score^b^	Diversity ranking^c^	Binding (in uM)
1	**SMADRAENL**	Rv1286	Q10600	268			>50
2	**SMAGRAGQL**	Rv3282	P96887	268			>50
3	**IMANRAQVL**	Rv3532	Q6MWW4	268			0,4
4	**KMNAKAATL**	Rv2911	Q7D6F2	263			15
5	**QMKFYAVAL**	Rv2119	O33254	260			1,5
6	**PMKRTALAL**	Rv0959	P0A5D7	259			18
7	**GMNVTAPAL**	Rv1617	O06134	257			8
8	**VMADRTRHL**	Rv0418	P96264	255			5
9	**WMCDRAVDL**	Rv2954c	Q50461	254			>50
10	**LMVVRALFL**	Rv3101c	P96293	254			nt
11	**EMVLRADQL**	Rv0191	O07435	253			>50
12	**DMLGRAGGL**	Rv3015c	O53262	253			>50
13	**MMKYLAFGL**	Rv0206c	O53657	246			0,4
14	**RMAATAQVL**	Rv2932	Q10978	237			2
15	**FLAADALVL**	Rv1047	P96354	185			>50
16	**VEAFRTRPL**	Rv1047	P96354	189			>50
17	**LMGAEADAL**	Rv1047	P96354	210			>50
18	**GLDSRAYRL**	Rv0146	O86321	212			>50
19	**GLDARAYRL**	Rv0145	P96356	212			>50
20	**PMAPLAPLL**	Rv3871	O69736	226			3
21	**EMKTDAATL**	Rv3874	P0A566	246			>50
22	**PMQQLTQPL**	Rv3873	Q79F92	205			>50
23	**PMADIAAAL**	Rv1253	Q11039	225			>50
24	**ALPPRAFEL**	Rv3428c	Q50700	212			0,27
25	**AMVNTTTRL**	Rv2074	Q10682	209			20
26	**LMGALAVVL**	Rv2075c	Q10683	211			21
27	**VSNLRTGKL**	Rv1967	O53968	208			>50
28	**TSADRAVVL**	Rv1973	O53974	203			39
29	**LMHYRGELL**	Rv1965	O53966	201			20
30	**NMMARGMDL**	Rv0221	P96403	201			>50
31	**LPAERAHEL**	Rv0222	P96404	202			>50
32	**VMMSEIAGL**	Rv1813c	P64889	348			13
33	**TMITFRLRL**	Rv1733c	P71991	373			2,4
34	**VMTTVLATL**	Rv1734c	P71992	348			1,8
35	**GMGMVGTAL**	Rv1737c	P71995	348			>50
36	**AMAGSIDLL**	Rv2006	Q10850	367			34
37	**EMLTSRGLL**	Rv1997	P63687	352			>50
38	**QMRACARRL**	Rv2004c	P0A5F9	210			1,5
39	**AMEYFRQVL**	Rv2006	Q10850	348			28
40	**SMFAAVQAL**	Rv2030c	O53475	375			5
41	**HMAQTLGSL**	Rv2030c	O53475	185			>50
42	**SMFAAVQAL**	Rv2030c	O53475	357			2,5
43	**EMGRAPLDL**	Rv2627c	O06185	373			>50
44	**RLPAKAPLL**	Rv1484	P0A5Y6		1,347		0,03
45	**VMAPLGPIL**	Rv1405c	P64841	NA			0,8
46	**QLAPGLQLI**	Rv1563c	Q10768		1,248		>50
47	**RLAPGGTTI**	Rv0068	O53613		1,238		>50
48	**RLANLLPLI**	Rv3189	O53335		1,231		32
49	**ILPSDAPVL**	Rv3823c	O07800	NA			0,2
50	**VLLPGLPYL**	Rv3847	P96230		1,208		8
51	**REPRRGPRL**	Rv0142	P96819		1,169	1	>50
52	**RLRPELAGL**	Rv3772	P72039		0,755	3	46
53	**RELPGRVLL**	Rv3094c	O05773		0,759	4	>50
54	**FLLPRGLAI**	Rv0056	P66315		0,867	5	>50
55	**VMATRRNVL**	Rv1518	Q50590		0,924	6	16
56	**RLGRLLNRI**	Rv3737	O69704		0,825	7	>50
57	**IEPRGAQAL**	Rv0171	O07415		0,985	8	>50
58	**DLPSRLGKI**	Rv3087	O53304		0,802	9	>50
59	**RPANLAFFL**	Rv1188	O50444	NA			1,3
60	**FMTRLGPLL**	Rv0035	Q7DAJ8		0,829	11	37
61	**RLASCRDAL**	Rv2761c	O33303		0,775	12	>50
62	**RMPPLGHEL**	Rv2997	O53244		1,19	13	4
63	**VMAPDAVRI**	Rv0324	O08446		1,014	14	31
64	**RSCPRLTIL**	Rv0894	P64749		0,895	15	>50
65	**RLARRARNI**	Rv1513	P71792		0,773	16	>50
66	**RLGLCALAL**	Rv3370c	O50399	NA			14
67	**VLPACLGIL**	Rv0206c	O53657		0,878	18	12
68	**VLRPGGHFL**	Rv1523	Q50584		0,767	19	1,8
69	**WLPPLLTNL**	Rv0249c	O53671		0,963	20	39

Peptides containing HLA-E binding motifs were predicted *in silico* using motif scores, discriminant analysis and diversity analysis. Peptide sequences are indicated as well as the proteins their originating proteins (Mtb Rv numbers and their Swissprot accession numbers). Binding was tested by HPLC size exclusion chromatography using recombinant human HLA-E and competition of a fluorescently labeled peptide [33]. Binding was calculated as the concentration (µM) of peptide required to reduce fluorescence intensity of the standard peptide with 50% (IC50).

*UniProtKB/ Swissprot accession number.

a Identified through “motif” score, i.e. weighting positions differently.

b Top five from a discriminant analysis: binder vs non-binder.

c Top Twenty-odd from diversity analysis of peptide sequences with high discriminant scores.

NA, not available.

DA often outperforms other approaches analyzing MHC-peptide binding because the method results in classification by class, from multiple data sources, regardless of the affinity measure used to identify binders [25]. Likewise, a peptide eluted from cell-surface MHC must obviously be able to bind, with reasonable affinity. Any peptide found, by overlapping peptide scanning, to be a T-cell epitope must, again, have bound to MHC prior to T cell recognition. Thus, all of these conceptually distinct ways of classifying peptides will yield equivalent definitions of binders versus non-binders. This allows a DA-based approach to rationalize a wide array of data. Other methods may weigh contributions from high-affinity peptides more than lower-affinity peptides, whereas DA will always weigh contributions equally according to class and will therefore generate sets of peptides with a greater probability of being active [25]. This prediction was complemented by diversity analysis to identify a set of peptide sequences which combined high potential binders with high diversity in terms of position-dependent physical properties as encoded using composite scores [26],[27]. Combination of all bioinformatics methods resulted in selection of 69 potential HLA-E binding peptides from the total Mtb H37Rv genome for detailed binding and immunological assays (Table 1).

### Binding assays

Peptides were synthesized and were tested for binding to recombinant human HLA-E*0103 in a competition assay, using a fluorescently labeled natural ligand [28] as the standard HLA-E binding peptide, and the predicted HLA-E peptides (n = 69) as competitors. Binding affinity (IC_50_<50 µM) was observed for 36 out of 68 peptides tested (53%), 18 peptides had a relatively high binding affinity for HLA-E (IC_50_<5 µM) (Table 1). As found for other HLA molecules as well [29], actual affinities determined in this biochemical cell free binding assay did not fully correlate with epitope prediction scores derived from bioinformatics (Table 1). Therefore we decided to test all 69 peptides for recognition by T-cells.

### T-cell proliferation is induced by predicted HLA-E binding Mtb peptides in the context of HLA-E

**Figure 1:**
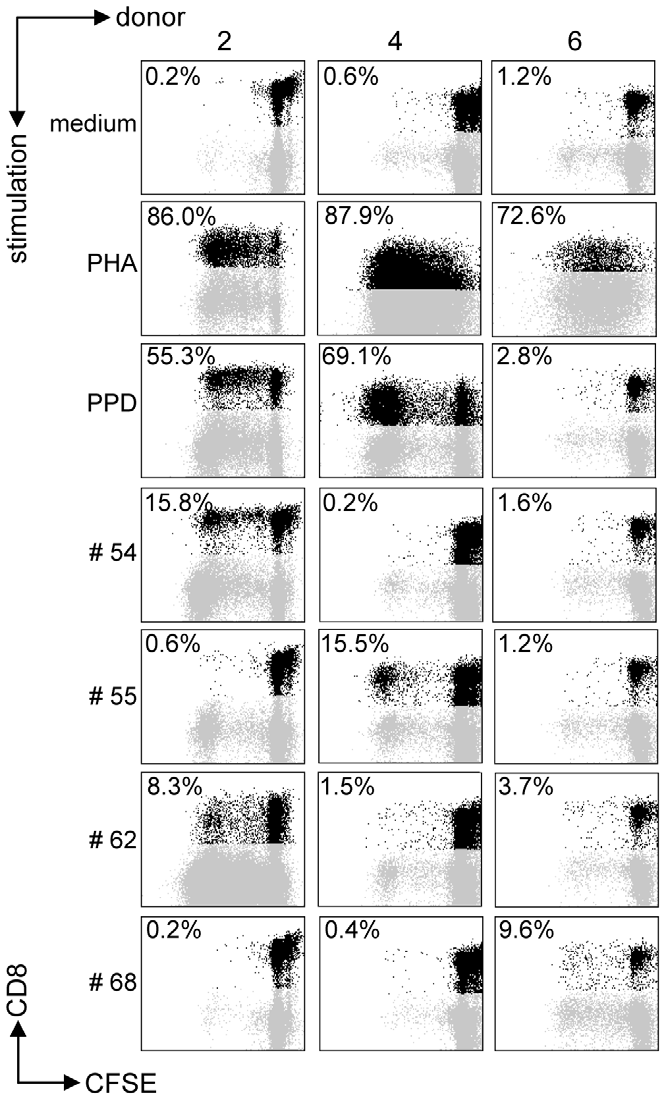
Predicted HLA-E binding Mtb peptides induce T-cell proliferation. CFSE-labeled PBMCs of 20 donors were stimulated with 10 µg/ml of peptide in the presence of IL-7, on day 7 low dose IL-2 was added and at day 10 proliferation was assessed by flowcytometry. Plots are gated on CD3^+^CD8^+^CD56^−^ cells in the lymphocyte gate, the percentage of proliferation indicated is within CD3^+^CD8^+^CD56^−^ cells. Peptide and donor numbers correspond to the numbers in Table 1.

All predicted HLA-E binding peptides were tested for their capacity to induce CD8^+^ T-cell proliferation as measured by CFSE dilution. Proliferating CD3^+^CD8^+^CD56^−^ T-cells were gated, thus excluding NK and CD4^+^ T-cells. We first assessed proliferation in PBMCs from healthy adult volunteers from the Netherlands who produced IFNγ (n = 10) or lacked IFNγ (n = 10) in response to Mtb-derived PPD. In all cases, unstimulated PBMC failed to proliferate whereas positive control mitogen (PHA) stimulated cells proliferated strongly (Figure 1). Proliferative responses were defined as >10% proliferating CD3^+^CD8^+^CD56^−^ T-cells based on CFSE dilution (see M&M section for details).

**Figure 2:**
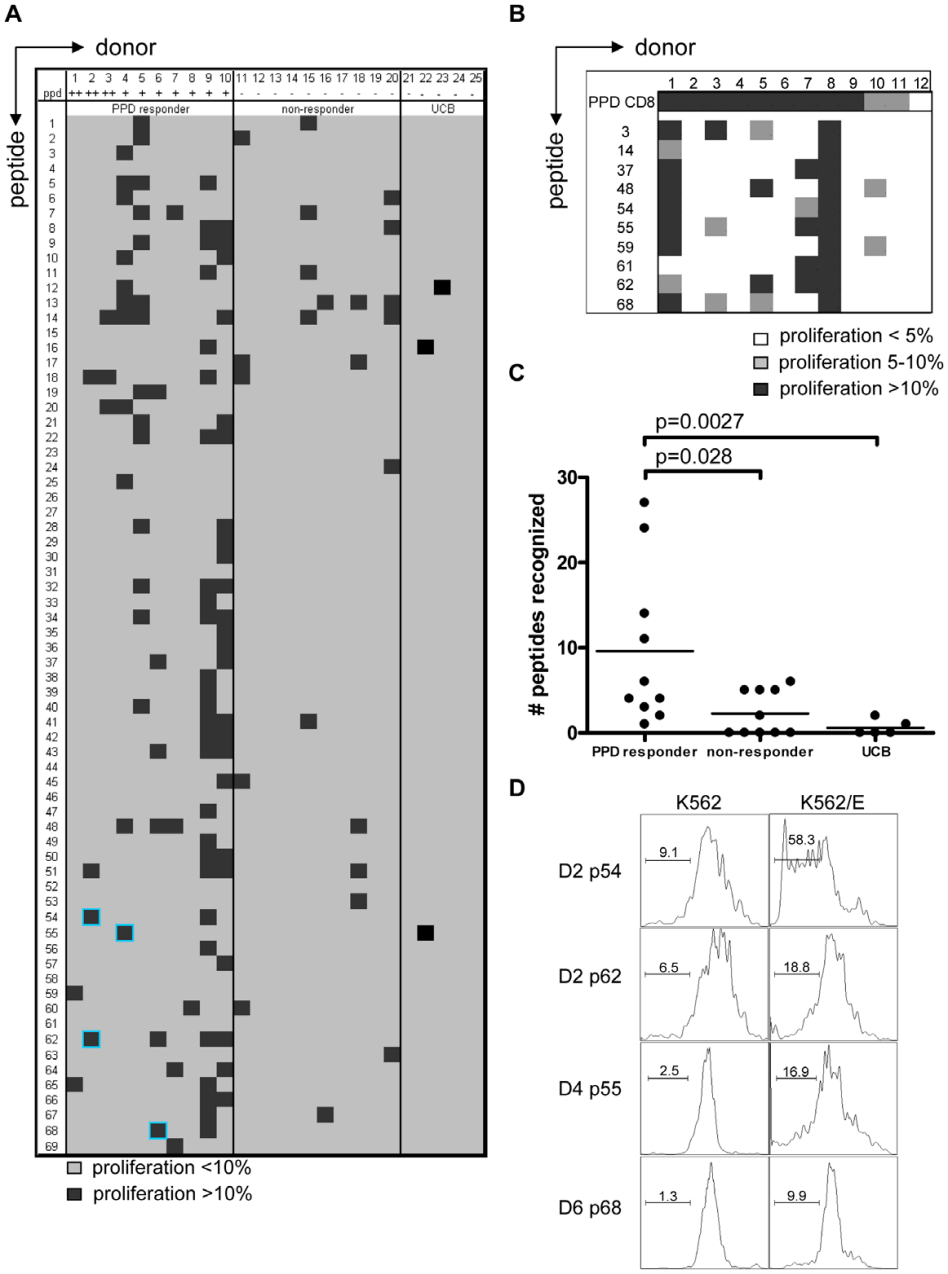
PPD responders and BCG vaccinated infants recognize predicted HLA-E binding peptides derived from Mtb. (A) Summary of all proliferation data from 10 PPD responders and 10 PPD non-responding donors. Proliferation was calculated using the following formula: Geometric mean of undivided population minus geometric mean of all cells. Subsequently the percentage was calculated by: (delta geo mean of sample- delta geo mean of negative control)/ delta geo mean of maximal proliferation (PHA). Samples with 10% specific proliferation or more were considered positive (black boxes). Blue highlights identify the peptides that were selected for detailed analysis. Umbilical cord blood (UCB) (n = 5) cell proliferation was assessed by the percentage of proliferating CD3^+^CD8^+^CD56^−^ cells and proliferation >10% was considered positive. (B) PBMCs from BCG vaccinated infants (n = 12) were tested in the same way as the cells from PPD responders for 10 selected peptides. Proliferation of 5–10% is indicated in grey and proliferation >10% in black. (C) Comparison of PPD responders, non-responders & cord blood samples for peptide recognition, i.e. the number of peptides recognized per donor (n = 10 for adults, n = 5 for cord blood). Analyzed using Students T-test with a p<0.05 considered significant. (D) CD8^+^ T cells were purified from PBMCs by magnetic bead separation of donors 2,4 and 6 and proliferation (CFSE) in response to peptide pre-pulsed K562 cells with (right plots) or without (left plots) HLA-E was assessed. Plots are gated on CD8^+^CD56^−^ cells and CFSE dilution is plotted.

Each of the 10 PPD-responding individuals tested recognized one or more of the 69 peptides (Figure 2A). There was significant inter-individual heterogeneity in the peptide repertoire recognized by the donors, and peptides that induced proliferation in one donor did not necessarily induce similar responses in other donors (Figure 1, 2A). Most donors recognized multiple predicted HLA-E binding epitopes (range 1–27, median 5). Conversely, 55/69 peptides (79%) were recognized by at least one PPD responder. Nine peptides were recognized by 3 independent adult donors and 2 peptides (#14 and 62) were even recognized by 4 PPD responding adult donors. Peptide sequence analysis did not reveal a structural motif associated with frequent recognition (data not shown). Compared to PPD responders, PPD nonresponsive donors showed much lower, though not undetectable frequencies of peptide responses (Figure 2A, 2C; p = 0.028). Five out of 10 non-PPD-responders did not recognize any of the peptides tested, whereas the other 5 donors recognized up to 6 peptides (overall range 0–6, median 1). One peptide (#13) which was recognized by 3 non-responder donors, revealed a broad presence in many mycobacterial strains, including *M. marinum*, *M. ulcerans*, *M. smegmatis* and *M. leprae* but likely also others, suggesting that T-cell proliferation may have resulted from cross-reactivity with ubiquitously present non-tuberculous mycobacterial species. Finally, there was no clear correlation between the number of peptides recognized and the levels of IFNγ produced following PPD stimulation in a 6-day lymphocyte stimulation test (data not shown).

Since some of the PPD non-responder donors responded to some of the test peptides, we decided to test cells derived from umbilical cord bloods (UCB) considering these to be immunologically most naïve, even though it has been demonstrated that also fetal exposure to mycobacteria can occur *in utero* [30]. UCBs recognized very few of the Mtb peptides with putative HLA-E binding Mtb peptides; three UCBs recognized none of the 69 peptides, one UCB recognized a single peptide and the remaining UCB recognized 2 individual peptides (Figure 2A & 2C). Taken together, these results suggest that the majority of responses against putative HLA-E binding Mtb peptides in adults most likely are the result of mycobacterial exposure or infection.

We next studied responses to a selection of the predicted HLA-E binding Mtb epitopes in a second, independent cohort of 10-week old South African infants that were routinely vaccinated with BCG at birth (n = 12). Proliferative responses in PBMC against predicted HLA-E binding peptides were observed in 6 out of 12 infants tested (Figure 2B). All 6 infants recognized 2 or more peptides. Six infants did not recognize any of the 10 peptides tested, despite detectable PPD responses. Infant #8 recognized all peptides tested but to a variable extent (range 23–53% of CD8^+^ T-cells). In line with the observations above the high affinity HLA-E binding peptides #62 and #48 were recognized with high efficiency. By contrast, peptide 61 was recognized by 2 BCG vaccinees, but not by any of the PPD responsive Dutch donors, suggesting possible differences in the induction of peptide specific responses between BCG vaccination and natural (NTM) exposure, or different ethnic groups, or both. In this setting, it was not possible to include control infants from the same geographic region since all infants routinely receive BCG within 24 hours after birth.

Taken together, the results indicate that CD8^+^ T-cells recognizing Mtb peptides containing a predicted HLA-E binding motif are detected preferentially and with appreciable frequencies in individuals responsive to PPD as well as in infants vaccinated with BCG. Of interest also is that the observed variation in peptide recognition patterns points to a relatively large array of HLA-E presented epitopes.

CD8^+^ T-cell proliferation against the predicted HLA-E binding Mtb peptides in polyclonal PBMC cultures might also (partially) have resulted from presentation by classical HLA class I molecules, due to possibly similar peptide binding motifs. To demonstrate that peptides presented by HLA-E can result in T-cell proliferation, K562 cells lacking endogenous HLA molecules but expressing only HLA-E were loaded with peptide and co-cultured with purified CD8^+^ T-cells. In this system no soluble peptide was present, thus also excluding T-T cell presentation [31]. CD8^+^ T-cell proliferation was observed in response to the selected peptides only when the target cells expressed HLA-E and had been peptide loaded. No such responses could be detected in the absence of HLA-E (Figure 2D). These data demonstrate that Mtb peptides presented by HLA-E can induce CD8^+^ T-cell proliferation.

### T-cell lines reactive with predicted HLA-E binding peptides have cytotoxic activity

To investigate the functional and phenotypical characteristics of the putative HLA-E binding peptide responding T-cells in more detail, we examined responses in 3 donors (2, 4, 6) against 4 peptides (54, 55, 62, 68) using peptide specific T-cell lines. Peptides and donors were selected based on the following criteria: 1. donors recognized multiple peptides; 2. there was clear peptide dependent T cell proliferation in that specific donor; 3. peptides were not recognized by PPD non-responders and 4. sufficient PBMCs available for detailed analysis. The 3 donors were fully typed for all HLA class I alleles, and prediction algorithms were run using HLA types of the donors and the selected peptides using the syfpeithi database (www.syfpeithi.de). The probability of peptide binding to HLA class Ia molecules was low for most donor-peptide combinations, further supporting that the observed CD8^+^ T-cell reactivity was mostly the result of HLA-E/TCR interactions. This is also supported by the above finding that K562 cells presenting Mtb peptides via HLA-E but not HLA class Ia can induce CD8^+^ T-cell proliferation.

**Figure 3:**
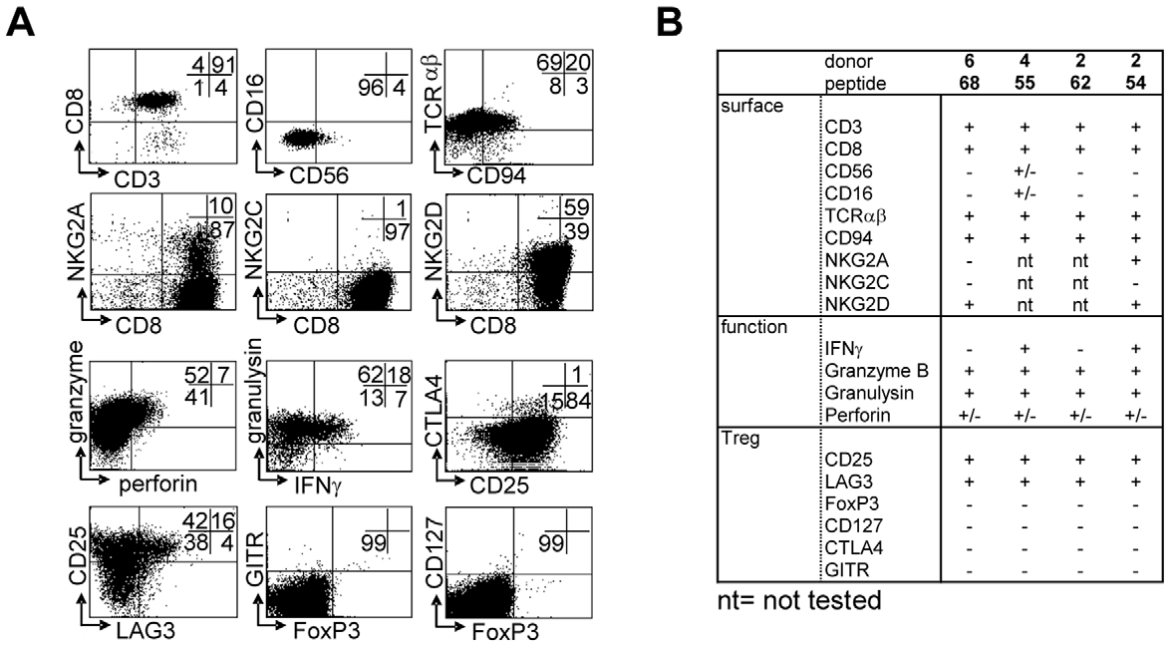
Surface-, functional- and Treg marker- phenotypes of predicted HLA-E binding peptide specific T-cell lines. (A) CD8^+^ T-cell lines were generated by peptide stimulation, and activated with αCD3/28 for 24 hours before staining. Brefeldin A was added for the last 16 hours only (3 µg/ml). Cells are gated on a lymphocyte gate combined with CD3 and CD8 gates. (B) Summary of phenotyping data from 3 donors and 4 peptides selected for further detailed analysis.

T-cell lines were generated by peptide stimulation and mostly displayed a CD3^+^CD8^+^CD56^−^CD16^−^TCRαβ^+^ phenotype, with a minority of CD56^+^ cells, as well as a subset expressing CD94 (Figure 3A). NKG2 family receptors were not abundantly expressed; of two lines tested neither expressed the activating NKG2C receptor. In contrast one line expressed the inhibitory NKG2A receptor on about 10% of its cells. Both lines however expressed NKG2D (Figure 3A, B). The majority of cells expressed “cell mediated cytotoxicity” markers: granzyme B, granulysin and low levels of perforin (Figure 3). Interestingly, several T-cell lines also displayed a phenotype partially compatible with regulatory T-cells, although not all classical markers were expressed: T-cells expressed high levels of CD25 and LAG-3 in the absence of CD127, but lacked FoxP3, GITR or CTLA4 (Figure 3A). All 4 T-cell lines had a remarkably similar phenotype, with the exception of IFNγ production, which was produced by only 2 out of 4 lines upon activation (Figure 3B).

**Figure 4:**
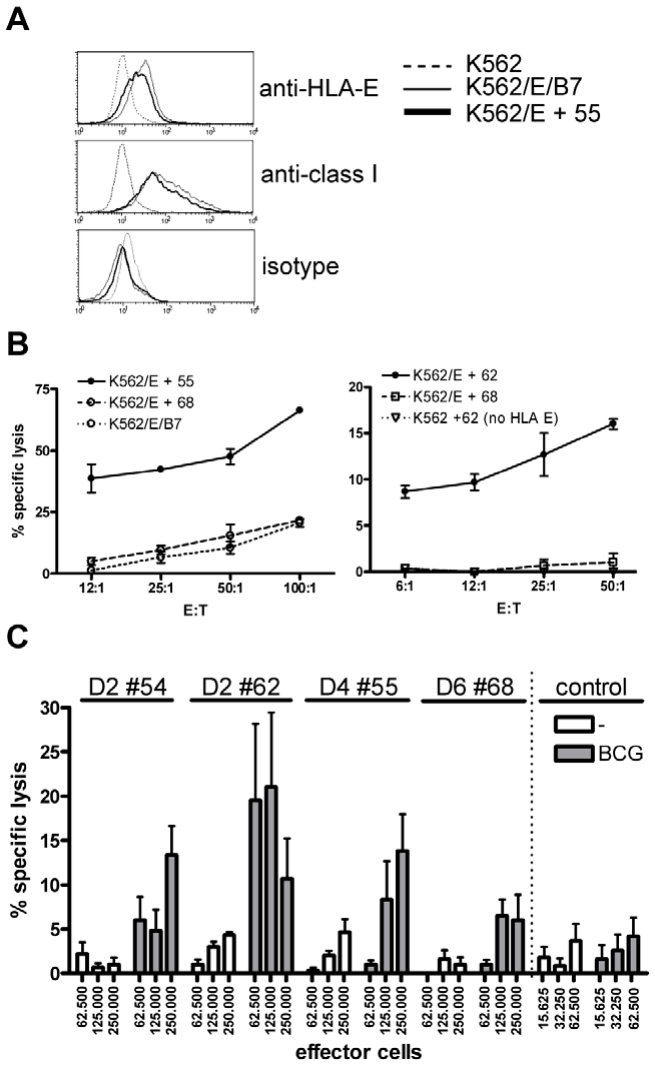
Predicted HLA-E binding Mtb peptide specific T-cell lines are cytotoxic. (A) HLA-E expression on K562 cell lines after peptide loading. Upper graph, untransfected K562 cells do not stain with anti-HLA-E (3D12) antibodies, whereas HLA-E transfected K562 cells expressing the natural HLA-E ligand (HLA-B7) or cells loaded with a Mtb derived HLA-E binding peptide (#55, see Table 1) express similar levels of HLA-E. Middle graph, similar staining is observed when an antibody against HLA class I (W6/32) is used, with equal expression levels of HLA-E in the presence of a natural ligand or an Mtb derived peptide. Bottom graph, the isotype control staining for 3D12 and W6/32 was negative on all cell lines tested. HLA-E expression was always confirmed before functional experiments. (B) Peptide specific T-cell lines were generated by stimulation with peptide in the presence of IL-7 and further expansion using IL-2. K562 cells expressing HLA-E were loaded with specific and control peptides, labeled with 1 µCi ^51^Cr and co-cultured with T-cell lines for 5 hours in different ratios before ^51^Cr release was measured. Data are expressed as % specific lysis. Left graph: T-cell line derived from donor 4 directed against peptide 55 (using the high affinity HLA-E binding peptide 68 or the natural B7 ligand as irrelevant control), right graph: T-cell line derived from donor 2 directed against peptide 62 (using peptide 68 on transfected K562, or peptide 62 on untransfected K562 as irrelevant controls). Representative of 4 lines derived from 3 donors. (C) Fully HLA-A,B,C mismatched adherent monocytes were infected with live BCG (MOI 5, overnight) before labeling with 1 µCi ^51^Cr. Cells were co-cultured with T-cell lines for 5 hours in 6-replicate cultures. Grey bars represent BCG infected monocytes whereas white bars represent uninfected monocytes. All HLA-E peptide restricted T-cell lines recognized BCG infected target cells. In contrast a CD8^+^ T-cell clone restricted to the male HY antigen presented in HLA-A2 did not lyse infected monocytes, whereas the natural ligand (B-cells expressing HY in the context of HLA-A2) was specifically lysed, resulting in up to 100% target cell lysis. Data are depicted as mean+standard error of the mean and represent multiple experiments.

Next, to determine the cytotoxic potential of the peptide generated T-cells in the exclusive context of HLA-E, we used K562 cells expressing HLA-E in the absence of any other HLA class I molecules. Untransfected K562 cells and HLA-E/HLA-B7 signal sequence transfected K562 cells were used as controls. Cells were loaded with peptide, labeled with ^51^Chromium and co-cultured with peptide induced effector T-cells. HLA-E expression on target cells was verified before each experiment. Co-expression of the natural HLA-E ligand, HLA-B7 signal sequence, induced HLA-E expression on the cell surface, detectable with both the HLA-E specific monoclonal antibody 3D12 as well as the pan HLA class I antibody W6/32, that also recognizes HLA-E (Figure 4A). Similarly, peptide loading of HLA-E transduced cells also induced equal surface expression detectable by both antibodies (Figure 4A).

The peptide 55 specific T-cell line specifically lysed HLA-E expressing target cells loaded with peptide 55 but not peptide 68. Control cells expressing HLA-E containing the HLA-B7 signal sequence were not recognized by these T-cells, thus confirming peptide specific recognition in the context of HLA-E (Figure 4B, left plot). The results were further supported by the observation that a T-cell line generated against peptide 62 specifically lysed K562 cells loaded with peptide 62 only in the presence of HLA-E (Figure 4B, right plot). Cytotoxicity was observed in all 4 T-cell lines and was consistently specific for the specific cognate Mtb peptide used to generate the T-cell line, and strictly required HLA-E expression. Taken together, these results demonstrate that T-cell lines generated against predicted HLA-E binding peptides are capable of recognizing and subsequently lysing target cells only when cognate peptide is presented by HLA-E, but not when irrelevant HLA-E binding peptides are presented.

Recognition of peptide loaded targets, however, does not demonstrate direct recognition of antigens on mycobacterium infected cells. To investigate this, HLA-A,B,C fully mismatched monocytes were infected with live *M. bovis* BCG, labeled with ^51^Cr and co-cultured with peptide specific T-cell lines. All 4 T-cell lines tested were able to lyse BCG infected, but not uninfected monocytes (Figure 4C), although the level of killing varied between different T cell lines. A control CD8^+^ cytotoxic T cell clone specific for the male HY antigen did not lyse BCG infected monocytes (Figure 4C), whereas it potently recognized the male HY antigen presented by HLA-A2 (data not shown). Importantly, these results demonstrate that T-cells generated against single HLA-E binding Mtb peptides can recognize live mycobacterium infected monocytes.

### Peptide specific T-cell lines also have regulatory capacities

**Figure 5:**
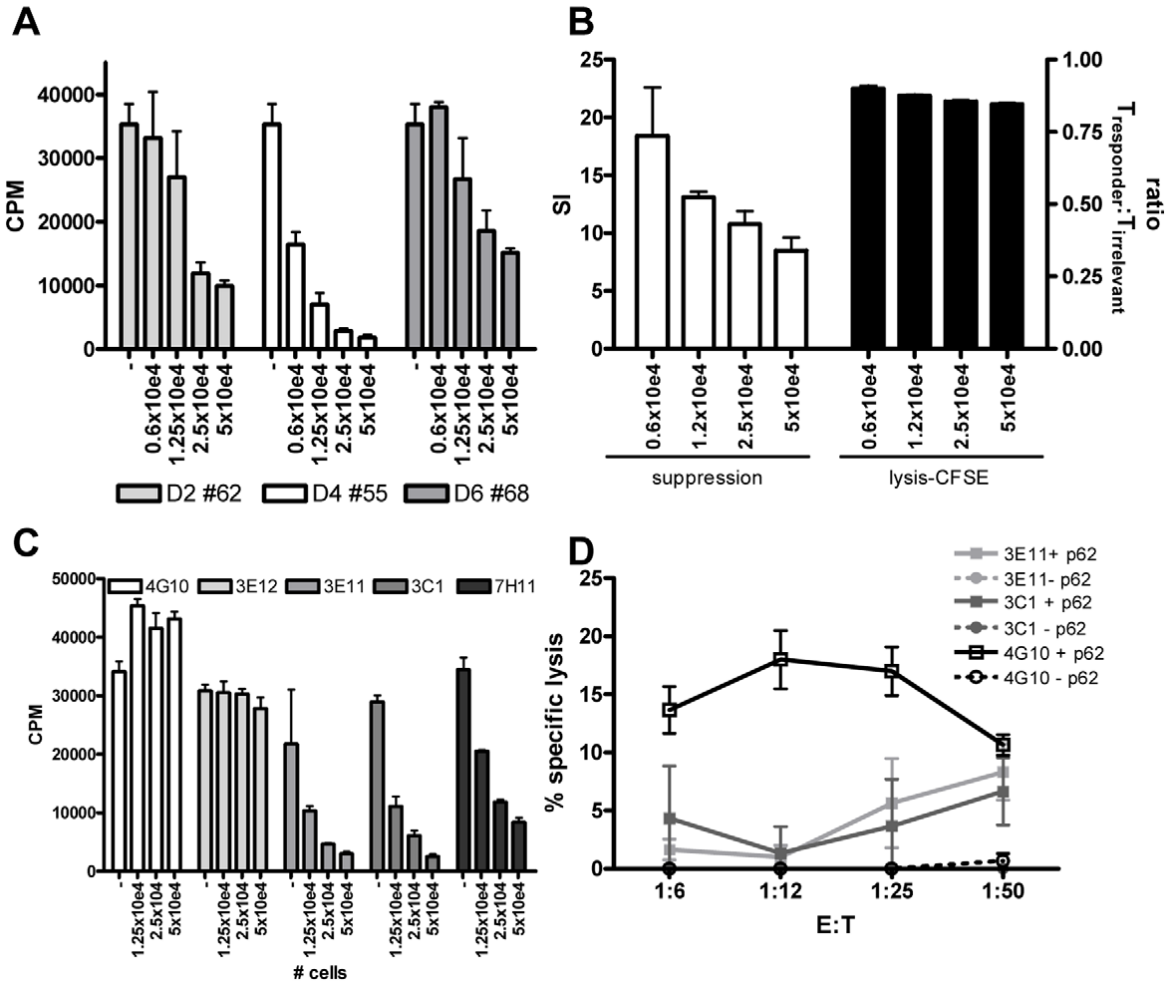
HLA-E binding peptide specific T-cell lines have immuno-regulatory activity. (A) Peptide specific T-cell lines were generated by stimulation with peptide in the presence of rIL-7 and further expanded with rIL-2. To investigate their potential capacity to inhibit CD4^+^ T-cell activity, they were co-cultured with a well characterized CD4 Th1 clone (Rp15 1-1 (1×10e4 cells per well)) proliferating to its cognate peptide (0.5 µg/ml) presented by HLA-DR3^+^ cells. Dose-dependent addition of peptide stimulated cells (ranging from 0.6–5×10e4 added T-cells) reduced proliferation of the Th1 clone as measured by [^3^H] TdR incorporation at day 3. T-cell lines generated from 3 donors directed against 3 different peptides are shown (donor 2 against peptide 62, donor 4 against peptide 55 and donor 6 against peptide 68), the T cell line from donor 2 against peptide 54 had a similar suppressive capacity (not shown) CPM = counts per minute. (B) To exclude that suppression was merely the consequence of lysis of the responder T-cells, we analysed cell survival in a CFSE labeling experiment. The responder T-cell clone Rp15 1-1 was labeled with a low dose of CFSE (0.005 µM), whereas a second, isogenic T-cell clone with a different peptide specificity and HLA-DR2 restriction (R2F10), was labeled with a high concentration of CFSE (5 µM). Both responder and irrelevant T-cell clones were HLA-E negative. They were then co-cultured with the HLA-E binding Mtb peptide specific T-cell lines (“Treg”) in the presence of the peptide (0.5 µg/ml) recognized by the responder clone Rp15 1-1 and HLA-DR3^+^ APCs. After 16 hours CFSE intensity was measured by flowcytometry. In the absence of added Tregs, similar numbers of responder and irrelevant T-cell clones were retrieved (ratio of 1). The addition of “Tregs” to peptide activated or control cultures also resulted in similar numbers of both responder and irrelevant T-cells, thus indicating that the responder clone is not lysed by the Tregs. Simultaneously a 3 day co-culture was performed and analyzed by [^3^H] TdR uptake. This experimental set-up revealed inhibition of proliferation of the responder clone. Addition of different numbers of Tregs did inhibit proliferation in a suppression assay but not the ratio of responder over irrelevant T-cell numbers in a CFSE intensity assay. (C) Clonal populations were obtained by limiting dilution of the T-cell line of donor 2 against peptide 62, all derived from 0.1 cells/well cultures. Clones were co-cultured in different ratios to Rp15 1-1 as described in (a) and 3H TdR incorporation measured. Three out of the 5 tested clones inhibited proliferation of the indicator clone in a dose dependent manner. (D) K562 target cells selectively expressing HLA-E were loaded with peptide 62 (10 µg/ml) before ^51^Cr labeling, followed by 5 hour co-incubation with T-cell clones and determination of ^51^Cr release. Clone 4G10 strongly lysed peptide loaded target cells, whereas 3E11 and 3C1 had moderate lysing capacity.

As mentioned above, the HLA-E/peptide specific T-cells also expressed several markers that have been associated with human CD8^+^ Tregs, notably CD25 and LAG-3 in the absence of CD127 [32], although they did not express FoxP3, GITR and CTLA4 (Figure 3). Interestingly, mouse T-cells restricted to the murine equivalent of HLA-E, Qa-1, display potent Treg activity [23]. For these reasons we decided to test whether T-cell lines specific for predicted HLA-E binding Mtb peptides also had immunosuppressive activity. T-cells were co-cultured with a well-characterized Th1 responder clone, Rp15 1-1 [32],[33], which recognizes Mtb hsp65 p3–13 peptide when presented by HLA-DR3. Using this previously reported, well-standardized read-out system [32], all 4 lines tested were found to suppress proliferation of the responder clone in a dose dependent fashion (Figure 5A).

To exclude the possibility that the reduced proliferation of the responder clone was the consequence of responder cell lysis, we performed a series of additional experiments. First, HLA-E expression was analyzed on the responder T-cell clone at various time points after peptide-APC induced activation. We did not observe any HLA-E expression on the responder T-cells (data not shown), thus excluding the possibility that HLA-E peptide recognition resulted in direct responder cell killing. However, since alternative mechanisms might be responsible, we labeled the Rp15-1-1 responder clone and an equal number of cells of an irrelevant T-cell clone (added to control for input cell numbers) pulsed with low and high doses CFSE, respectively, and added both cells into the co-culture suppression assay. After 16 hours, a time point prior to division of responder cells, but sufficient for cytolysis to occur (typically detectable after 4–5 hours) fluorescent intensities of responder and irrelevant control T-cell clones were not altered by the addition of HLA-E/peptide induced Tregs, irrespective of the presence of the cognate peptide of the responder clone. Nevertheless, the addition of Tregs exerted strong suppression of [^3^H] TdR incorporation after 72 hrs. These control experiments indicate that HLA-E/peptide induced Tregs inhibited proliferation of, but did not lyse, responder T-cells (Figure 5B).

As shown above, the HLA-E binding Mtb peptide specific T-cell lines had both cytotoxic and regulatory activity. Dual functionality has been observed in polyclonal lines [32], but it remains unclear whether these functions are exerted by the same or different T-cell subpopulations. To investigate single vs dual functionality, single cell derived T-cell lines or ‘clones’ were derived from limiting dilution of a polyclonal T cell line. Three out of the 5 T-cell clones obtained after expansion had potent regulatory activity (Figure 5C), whereas the other 2 had no such activity. These findings imply that only a subset of HLA-E/peptide induced T cells has regulatory properties. The results also exclude that the experimental protocols used to generate T-cell lines skewed towards expansion of cells with regulatory activity.

Next, 3 of the 5 T-cell clones were tested for cytotoxic activity towards peptide loaded HLA-E^+^ target cells. One clone (4G10) which lacked regulatory activity displayed potent cytotoxic activity, whereas 2 other clones (3E11, 3C1) which had potent regulatory activity had only moderate cytolytic activity (Figure 5D). This demonstrates that cytotoxic and regulatory activity can be, but are not necessarily mediated by the same HLA-E/peptide induced cells (Figure 5C and 5D).

### Membrane-bound TGFβ is involved in suppression of proliferation

Since suppression was not mediated by cellular cytotoxicity, alternative possibilities were explored. First, supernatants were generated by stimulating all 4 T-cell lines with cognate and control peptides in the presence and absence of HLA-E expressing APCs, and by stimulation with αCD3/28 (to allow maximal cytokine production). Supernatants were collected at various time points, and then transferred to stimulated responder T-cells. None of the added supernatants was able to inhibit the responder T-cell clone, whereas the physical presence of the regulatory T-cells was able to do so (data not shown), indicating that cell-cell contact is required for suppression. Several molecules have been described that can mediate cell-cell contact dependent suppression, including CTLA4 [34], GITR [35] and, more recently, membrane bound TGFβ1 [36],[37]. Since our regulatory T-cell-lines did not express significant levels of CTLA4 or GITR (Figure 3A), these molecules were unlikely to be involved, such that we decided to examine whether membrane bound TGFβ (mTGFβ) might be involved.

**Figure 6:**
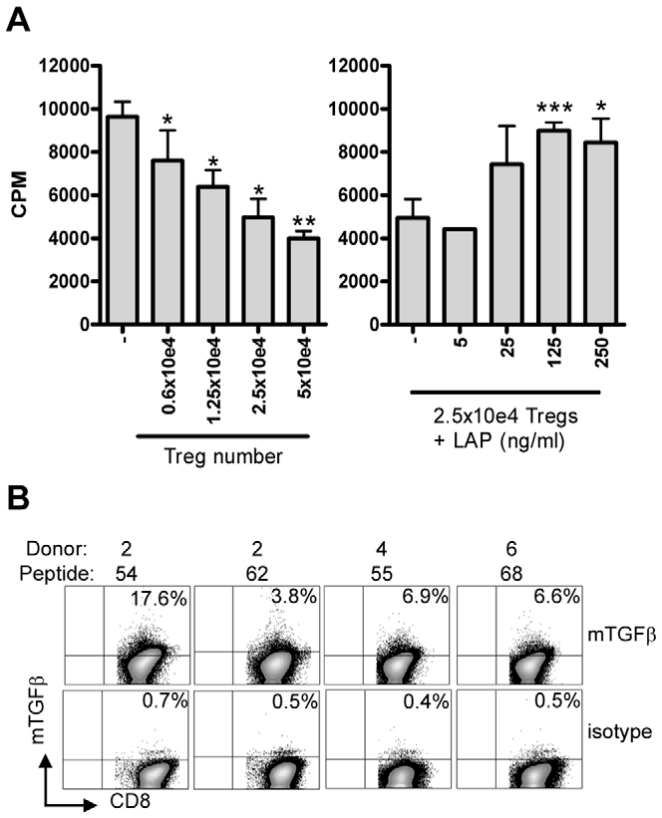
Suppression is mediated by membrane-bound TGFβ. (A) Co-culture assay of Treg line (donor 4, peptide 55) with responder T-cell clone (Rp15 1-1) in the presence of 0.5 µg/ml cognate peptide and HLA-DR3^+^ APCs results in inhibition of proliferation ([^3^H] TdR uptake; left graph). Right graph: Addition of LAP in this setting in the presence of a fixed number (2.5×10e4) of Tregs resulted in a dose-dependent abrogation of suppression. Experiment representative of 4 lines in 3 different experiments. *: p<0.05, **:p<0.01, ***: p<0.001 (Students T-test). (B) Surface staining of membrane bound TGFβ. T-cell lines were stained for the presence of membrane bound TGFβ using the TB21 monoclonal antibody, gated on CD25^+^ cells. mTGFβ staining was observed on all 4 lines, although to a variable extent, the isotype control was used to place markers and was negative for all lines (bottom).

The function of active TGFβ can be inhibited by addition of latency associated peptide (LAP), which reverts active TGFβ to a latent, inactive form [38]. Addition of recombinant LAP to co-culture assays indeed resulted in reversal of suppression, strongly implicating a functional involvement of TGFβ in inhibition of T-cell proliferation (Figure 6A). Addition of LAP to the indicator clone only resulted in an increased proliferation of maximally 20% (data not shown), similar to previous studies [36]. However the increased proliferation observed upon addition of LAP to co-cultures of Tregs and indicator clones resulted in an increase of proliferation over 40% supporting a specific reversal of suppression.

Since suppression was contact dependent and acidified supernatants did not contain any TGFβ1 as measured by ELISA (data not shown), we assumed that TGFβ was membrane bound. Cell surface staining using a TGFβ1 specific monoclonal antibody indeed revealed the presence of mTGFβ at the surface of activated T-cell lines (Figure 6B).

Taken together, these data demonstrate that mTGFβ is expressed on HLA-E restricted, Mtb peptide reactive T-cell lines, which have regulatory activity, and that its functional inhibition abrogates, in part, the suppression by these T-cells. Thus, Mtb derived peptides presented by human HLA-E are recognized by CD8^+^ T-cell populations that can exert both cytotoxic and immunoregulatory functions, the latter involving membrane bound TGFβ.

## Discussion

We describe to the best of our knowledge for the first time, *Mycobacterium tuberculosis*-derived peptides that can be presented by HLA-E molecules and are recognized by human CD8^+^ T-cells. These observations significantly extend our knowledge of the repertoire of epitopes and human antigen presentation pathways in mycobacterium specific host immunity, complementing current knowledge on the well established classical HLA class Ia, class II and CD1 group 1 presentation molecules and presentable (peptide and non-peptide) ligands [39]. Human CD8^+^ T-cells from PPD-responsive adults and BCG-vaccinated infants recognized newly identified Mtb peptides with predicted HLA-E binding motifs, resulting in T cell proliferation. The responding cells had cytotoxic activity, and lysed target cells in a peptide-specific and HLA-E dependent fashion, strongly suggesting peptide/HLA-E cognate recognition via the TCR. Moreover, these epitopes likely are also recognized during infection since live mycobacterium infected monocytes were lysed by HLA-E/peptide stimulated effector T cells. In addition to their cytolytic capacity, HLA-E/peptide reactive T-cell lines also had strong immunoregulatory properties, since they inhibited proliferation of unrelated responder T-cells. This suppression was dose-dependent, required cell-cell contact and was mediated, at least in part, by membrane bound TGFβ1 (mTGFβ1). Thus, the human CD8^+^ T-cell lines described here have dual functionality, in that they are able to lyse antigen loaded target cells, which may be linked to protective effector mechanisms in controlling intracellular infection, and to exert immunoregulatory activities. T cell lines or ‘clones’ derived from limiting dilution assays demonstrated that cytotoxic and regulatory activity can be, but are not necessarily mediated by the same HLA-E/peptide induced cells.

It has previously been described that antigens from *Salmonella typhi* [9] and cytomegalovirus [7] can be presented by human HLA-E molecules. In the case of Mtb, 2 HLA-E restricted T-cell clones have been reported, yet the precise peptides from Mtb that were recognized (which were present in Mtb-DC-conditioned medium) remain unknown. It is also unknown to what extent these T-cell clones might represent the global response against Mtb in humans [13]. In general, the number of potential HLA-E peptide epitopes identified in the literature and the nature of T-cells recognizing them has been very limited. Interestingly, HLA-E is enriched in the Mtb phagosome compared to regular HLA class I molecules (HLA-A2), suggesting that HLA-E may have unique functions in presenting phagosomal antigens, which is particularly relevant to Mtb since it resides in immature phagosomes [40].

To the best of our knowledge, the current study describes the first large scale analysis of pathogen derived, putative HLA-E binding peptides, revealing a significant contribution of HLA-E dependent peptide presentation in pathogen recognition. Moreover, our study represents the first genome wide pathogen screening based on bioinformatic algorithms, predicting peptides with a potential HLA-E binding motif. In our experiments, epitope prediction scores, peptide binding affinity measurements, T-cell recognition and functional profiling were used to guide peptide selection. Interestingly, some of the peptides studied were not capable of competing with the high affinity natural ligand in the peptide/HLA-E competition assay, regardless of being efficiently recognized by T-cells. Thus, for prediction of HLA-E binding peptides, some caution seems warranted when selection is based solely on HLA-E binding affinity in biochemical, cell free binding assays. In addition, the results show, in line with literature on peptides bound by HLA-class Ia and II molecules, that high affinity peptide binding does not necessarily translate into high efficiency T-cell recognition. This may be due to the lack of processing, induction of tolerance or alternative binding registers not involved in TCR engagement. Although the subsequent rounds of peptide prediction (initially motif scores were used, followed by discriminant analysis and finally diversity ranking) and binding aimed to improve the prediction algorithms, this might not have been unequivocally successful: although numbers are sometimes small, the data suggest that peptides derived from all 3 prediction rounds bind equally well and are recognized by T-cells to a similar extent. Thus the motif was not further improved and dedicated studies should be performed for motif optimization.

The abundant recognition of Mtb peptides with an HLA-E binding motif, points towards a contribution of non-classical HLA molecules to pathogen specific immunity in general and perhaps more particular for mycobacteria in view of Mtb's phagosomal localization [40]. We hypothesize that functional effector T-cells induced by HLA-E binding Mtb peptides contribute to pathogen clearance *in vivo*. Peptides containing a HLA-E binding motif were also recognized by infants following BCG vaccination, suggesting that antigen presentation of mycobacterial peptides *in vivo* can result in activation of CD8^+^ T-cell immunity in the context of HLA class Ib molecules. This is further supported by the observed lysis of *M. bovis* BCG infected monocytes by the HLA-E/peptide stimulated T-cell lines, suggesting specific recognition of mycobacterium infected target cells by HLA-E restricted T- cells.

Since HLA-E is a highly conserved molecule, one might have expected more uniform recognition patterns of synthetic peptides derived from Mtb between different individuals. However, as shown in the present study, peptide recognition is rather diverse and none of the peptides is recognized by all responsive or vaccinated donors. Thus, the repertoire of HLA-E binding peptides from Mtb -and perhaps also other antigens- may be larger than initially anticipated. Nevertheless, a small number of peptides (e.g. #62) was recognized by over 30% of the PPD responsive donors in 2 quite different cohorts. It remains also possible that a proportion of the response could have been due to proliferation in response to classical HLA class Ia family members that can bind the same peptides. However, in the donors tested the motifs of the recognized peptides did not conform to any of the classical HLA-A and B allele specific peptide binding motifs.

Some peptides were also recognized by donors who did not have detectable T-cell responses to PPD *in vitro*. We do not have detailed information about these (anonymous bloodbank) donors and can therefore not exclude that some donors may have encountered mycobacteria in the past [41]. The fact that certain peptides in these donors were more able to induce CD8^+^ T cell responses compared to PPD might be due to their preprocessed nature, facilitating high efficiency antigen presentation and/or to their higher molarity, since proteins and peptides all were tested at 10 µg/ml concentrations, regardless of their molecular mass. Umbilical cord blood samples, however, displayed lower levels of proliferation, suggesting that sensitization to (environmental) (myco)bacteria had caused proliferation in non-responders. Of note, some of the peptides recognized by PPD non-responder donors are not unique to Mtb, but are also present in environmental mycobacteria and other bacteria, such that the observed responses may have been the result of cross-induced immunity. The impact of environmental (myco)bacteria on induction of T-cell immunity needs further study [41].

CD8^+^ T-cells that recognize HLA-E restricted *S. typhi* derived peptides can produce IFNγ and lyse infected target cells, indicating a role in host defense [9]. Such a functional contribution of HLA class Ib genes to host defense is interesting, particularly in view of the low genetic polymorphism of HLA class Ib genes. Murine studies have shown that the HLA-E homologue Qa-1 can not only be recognized by CTLs but also induce Tregs [42]. Qa-1 knockout mice had increased CD4^+^ T-cell responses upon infection and vaccination, due to the lack of Qa-1 restricted CD8^+^ Tregs [42]. Indeed, our CD8^+^ T-cell lines were also able to inhibit T-cell proliferation via membrane bound TGFβ. In theory, both functions may be the property of a single cell population or alternatively be expressed by 2 subpopulations. Our preliminary experiments show that single-cell derived ‘T-cell clones’ can possess either both regulatory and cytotoxic dual activity or single functionality. This demonstrates that cytotoxic and regulatory activity are not necessarily mediated by, yet can be exerted by the same cellular populations.

The role of Tregs in TB infection is debated although some recent studies in murine infection models show that they preclude efficient pathogen clearance and are therefore harmful to the host [43],[44]. Although direct evidence is lacking, indirect evidence indicates that Tregs are involved in active disease in humans as well. First, CD4^+^ and CD8^+^ Tregs were increased at the site of infection, both in mycobacterium induced granulomas [32],[45] and at sites of extrapulmonary TB compared to the circulation [45]–[48]. Secondly, the frequency and number of Tregs was increased in the circulation of patients with active TB compared to controls, and normalized after treatment [44]–[47]. Depletion of Tregs *in vitro* increased IFNγ production in response to mycobacterial antigens [47],[48]. Thus the presence and activity of Tregs is associated with (and a biomarker of) active TB disease. *In vitro* stimulation with peptides containing a HLA-E binding motif resulted in the induction of functionally active regulatory T-cells. If similar activity occurs following vaccination with putative HLA-E binding Mtb peptides, these Tregs may regulate rather than mediate pathogen eradication. In this context, it is interesting to note, however, that it was recently described that Treg activity can also contribute to host immune protection against infectious agents, by allowing a timely entry of effector cells during viral infections [49]. The relative contribution of these different functional T-cell properties remains to be clarified in future studies.

Another open question is whether peptide recognition in the context of HLA-E is an exclusive gateway for CD8^+^ Treg induction. To decipher if HLA-E preferentially activates T-cells with regulatory properties compared to classically restricted T-cells, more detailed quantitative comparisons will be needed between classically and non-classically restricted T-cells regarding the frequency of Tregs within these populations. Until these analyses have been completed we can only conclude that HLA-E restricted cells can have regulatory properties. Previously we have described CD8^+^ Tregs upon *in vitro* stimulation with live BCG [32], but we have not confirmed or excluded a role for HLA-E in antigen presentation to these CD8^+^ Tregs. However these cells had a different phenotype and used a different mechanism of suppression (i.e. via CCL4), thus suggesting that the CD8^+^ T(reg)-cell response to mycobacteria is heterogeneous and includes multiple subsets with regulatory activity.

At this stage, we can only speculate why T-cells that recognize foreign, pathogen-derived peptides in the context of HLA-E might have dual (cytolytic and regulatory) functions. The low level of variation in HLA-E proteins suggests a similarity with pattern recognition in innate immunity, possibly with a primary default effector T-cell response as a consequence. Natural ligands of HLA-E are signal sequences derived from other HLA class I molecules; in all cases the immune system aims to avoid harmful immunity to these self-antigens. HLA-E is amongst the few HLA molecules expressed in the human throphoblast, which invades the maternal part (the decidua) of the placenta [50]. Expression of HLA class I alleles is necessary to evade immune surveillance by NK cells, but recognition of allo-antigens by the maternal immune system is undesirable for the fetus. Increased Treg numbers are observed in the placenta, mostly at the site of feto-maternal contact [51],[52], perhaps as a result of HLA-E presented peptides. Pathogen derived peptides might have hijacked this mechanism in order to be able to induce pathogen specific Tregs that can down regulate host immunity. This balance between effector and regulatory immunity in the context of HLA-E might allow partial clearance of pathogen from the host, thus providing sufficient levels of protection while avoiding excessive inflammation and pathology, but at the expense of pathogen persistence and chronic infection. Future studies need to dissect this “primordial” host immune response pathway, and to determine the relative importance of effector vs. immunoregulatory activities within the HLA-E based antigen presentation system in infection and other human diseases.

## Materials and Methods

### Ethics statement

Human participation in this research was according to the U.S. Department of Health and Human Services and good clinical practice guidelines. This included protocol approval by the Leiden University Medical Center Ethics Committee and the University of Cape Town research Ethics Committee and written (parental) informed consent by all donors. Anonymous buffy coats from healthy blood bank donors were only used if donors had consented scientific use of blood products.

### Prediction of HLA-E binding Mtb peptides

A set of open reading frames corresponding to all (predicted) proteins from the Mtb (H37Rv) genome was scanned using a semi-quantitative scoring matrix to search for nonameric peptides with the potential to bind HLA-E. The matrix was adapted by a motif change and inclusion of a quantitative matrix, derived from the results of Miller *et al* [20]. The top scoring set of peptides was combined with a set of legacy peptides of known provenance and a set of other available peptides as potential negative controls (peptides 1–43). The resulting set of peptides was synthesized and tested in *in vitro* binding assays. This data was used to create a quantitative discriminant model [25]; ranking peptides in terms of their likelihood of binding HLA-E, yielding a second set of peptides (peptides 44–50). The ranked set of Mtb peptides was then subjected to diversity analysis using Z score descriptors for each position [26],[53],[54] to generate a third set of peptides which were synthesized and tested for HLA-E binding and immunogenicity (peptides 51–69).

### Peptide synthesis

Peptides were made on a Syro II peptide synthesizer (MultiSyntech, Witten, Germany) using TentagelS AC resins (Rapp, Tübingen, Germany) in combination with Fmoc chemistry [55],[56]. The purity of the peptides was checked on reverse phase C18 HPLC (Vydac 218TP5415, Grace, Deerfield, IL, USA). As the standard peptide VMAPC(Fl)TLLL was used. This peptide is derived from the human HLA-B*0801 leader peptide VMAPRTLLL. Fluorescence labeling of the cysteine in the precursor peptide was performed with 4-(iodoacetamido)fluorescein (Fluka Chemie AG, Buchs, Switzerland) in a mixture of 250 µl Na-phosphate buffer 0.15 M, pH 8.0 and 150 µl acetonitrile [55].

### HLA-E-peptide binding assay

Recombinant HLA-E*0103 (kind gift of Dr. V.M. Braud, Université de Nice-Sophia, Valbonne, France) was overexpressed in *E. coli* and purified as described previously for HLA-A*0201 [57], and then dissolved in 8M urea and stored in stock solutions (50 µM) at −20°C until use. The integrity of the protein was confirmed by TOF-MALDI mass spectrometry. Human β2-microglobulin was purchased from Sigma (St. Louis, MO) and dissolved in H_2_O.

HLA-E*0103 was titrated in the presence of 100 fmol fluorescent standard peptide to determine the recombinant HLA concentration necessary to bind 20–50% of the total fluorescent signal. All subsequent inhibition assays were then performed at this concentration. HLA-E*0103 was incubated in 96-well plates (polypropylene, serocluster, Costar) at RT (pH7) for 24 h with 15 pmol β2M and 100 fmol fluorescent labeled standard peptide in assay buffer (100 mM Na-phosphate, 75 mM NaCl, 1 mM CHAPS), protease inhibitor mixture (1 µM chymostatin, 5 µM leupeptin, 10 µM pepstatin A, 1 mM EDTA, 200 µM pefabloc) and 2 µl of the peptides of which HLA-E binding capacity was to be determined. As a standard peptide VMAPC(FL)TLLL was used. The HLA-peptide complexes were separated from free peptide by gel filtration on a Synchropak GPC 100 column (250mm × 4.6mm; Synchrom, Inc., Lafayette, Indiana). Fluorescent emission was measured at 528 nm on a Jasco FP-920 fluorescence detector (B&L Systems, Maarssen, The Netherlands). As HPLC running buffer, assay buffer containing 5% CH_3_CN was used. The percentage of peptide bound was calculated as the amount of fluorescence bound to MHC divided by total fluorescence. The concentration of peptide yielding 50% inhibition was deduced from the dose-response curve. Each peptide was tested in at least two separate experiments.

### Donors

Anonymous buffy coats were collected from healthy blood bank donors (Dutch, adults) that all had signed informed consent. No clinical information is available for the donors other than that they were healthy, and had no chronic viral infections or other contraindications for donating blood. BCG in The Netherlands is only administered to people at risk for TB exposure and the TB incidence in the Netherlands is extremely low, such that the vast majority of our donors (>95%) is highly unlikely to have been vaccinated with BCG, or to have had exposure to Mtb. PBMCs were isolated by density centrifugation and directly tested in a lymphocyte stimulation test to analyze their reactivity to Mtb derived antigens. PBMCs (5×10e5/well) were stimulated with PHA (2 µg/ml, Remel, Oxoid, Haarlem, The Netherlands) and PPD (5 µg/ml, Statens Serum Institute, Copenhagen, Denmark) in Iscove's modified Dulbecco's medium (IMDM, Invitrogen, Breda, The Netherlands) containing 10% pooled human serum. After 6 days supernatants were tested in an IFNγ ELISA (U-CyTech, Utrecht, The Netherlands). IFNγ production ≥100 pg/ml was considered a positive response [58].

Ten donors that had IFNγ responses (≥100 pg/ml) to PPD stimulation as well as 10 donors that did not respond were selected for the immunogenicity screening of peptides with predicted HLA-E binding motifs. The average IFNγ production in response to PPD was 2067 pg/ml, median 1213 pg/ml (range 275–10,000 pg/ml).

Anonymous umbilical cord blood (UCBs) cells were provided by Dr. S. Scherjon (dept. of Obstetrics, Leiden University Medical Center), all were derived from full term-pregnancies and delivery by caesarian section.

As a second group we analysed BCG vaccinated infants, to examine whether BCG vaccination could elicit response against predicted HLA-E binding epitopes. Similar proliferation experiments were performed using cells from 10 week old infants (n = 12, from South Africa, with informed consent from their parents) that had received BCG vaccination at birth. Peptides were selected after the screening in healthy Dutch adults was completed and included the 4 peptides selected for detailed analysis, the top 3 peptides recognized by Dutch donors and 3 high affinity HLA-E binding peptides, irrespective of recognition by Dutch donors.

### Analysis of T-cell proliferation induced by predicted HLA-E binding Mtb peptides

PBMCs were CFSE labeled (5 µM, Invitrogen), 1×10e5 cells were stimulated with predicted HLA-E binding peptides at a concentration of 10 µg/ml in IMDM with 10% human serum in the presence of 5 ng/ml IL-7 (Peprotech, Rocky Hill, NJ). Positive and negative controls were included in each assay and included PHA (2 µg/ml), PPD (5 µg/ml), ESAT-6 and CFP-10 (10 µg/ml each) or culture medium only. On day 7 of culture, IL-2 was added to a final concentration of 10 U/ml (Cetus, Emeryville, CA). On day 10, supernatants were collected and stored at −20°C. Cells were harvested, replicates (n = 6) pooled and stained using CD3-PerCP, CD8-APC and CD56-PE (all BD Biosciences, Alphen aan de Rijn, The Netherlands) before acquisition on a FACS Calibur flowcytometer using CellQuest Pro software (BD Biosciences) or on a LSRII flowcytometer using FACS Diva software (BD Biosciences).

To analyze proliferation, cells were gated on lymphocytes, followed by gating on CD3^+^CD8^+^CD56^−^ cells. The percentage of proliferation was calculated using geometric means by subtracting the geometric mean of all cells from the geometric mean of the undivided population. Subsequently the percentage was calculated by: (delta geo mean of sample- delta geo mean of negative control)/ delta geo mean of maximal proliferation.

To demonstrate that putative HLA-E binding Mtb peptide directed CD8^+^ T-cell proliferation in PBMC cultures can indeed be induced by HLA-E peptide presentation we performed the following experiment. CD8^+^ cells were purified from PBMCs from 3 donors (2,4 and 6) by positive selection using magnetic beads (MACS, Milteny Biotec, Auburn, CA) and labeled with CFSE (5 µM, Invitrogen), the CD8^−^ fraction was irradiated at 30 Gy and added to the culture as feeders. Peptides (25 µg/ml) were loaded onto K562 cells with or without HLA-E*0103 (kind gift of Dr. E. Weiss, Ludwig-Maximilians-Universität, Munich, Germany) [59] during an overnight period at 26°C, followed by stabilization at 37°C for at least 2 hours. After washing to remove any free peptide, peptide loaded target cells were irradiated at 50 Gy and HLA-E expression was checked by FACS. CFSE labeled CD8^+^ cells (1×10^4^) were co-cultured with peptide loaded K562 cells (5×10^3^) with or without HLA-E in the presence of irradiated self CD8^−^ cells (2,5×10^4^) in 96 well roundbottom plates (12 wells per condition). Cells were cultured in IMDM+10% human serum supplemented with 5 ng/ml IL-7 and with costimulatory antibodies (CD28 (1 µg/ml, CLB, Amsterdam, The Netherlands) & CD49d (1 µg/ml, BD Biosciences)). On day 7 of co-culture, IL-2 was added to a final concentration of 10 U/ml (Cetus, Emeryville, CA). On day 10, cells were harvested and stained using CD3-Pe-Cy7, CD8-APC and CD56-Alexa 700 (all BD Biosciences, Alphen aan de Rijn, The Netherlands) before acquisition on a LSRII flowcytometer using FACS Diva software (BD Biosciences). For analysis, cells were gated on CD8^+^CD56^−^ and CFSE proliferation was analysed.

### Generation of T-cell lines & clones

PBMCs were stimulated with single predicted HLA-E binding peptides (10 µg/ml) in IMDM with 10% pooled human serum and 5 ng/ml recombinant human IL-7. On day 6 of culture IL-2 was added to a final concentration of 25 U/ml.

CD8^+^ T-cells were isolated from peptide induced T-cell lines at day 13 by magnetic bead separation (MACS, Milteny Biotec, Auburn, CA). For restimulation, feeder cells were pre-pulsed with 10 µg/ml of the specific peptide for 4 hours, washed and irradiated (30 Gy), 1×10e5 prepulsed feeder cells were added to 2×10e4 T-cells in the presence of 125 U/ml IL-2 (Cetus) in IMDM (Invitrogen) with 10% pooled human serum.

T-cell clones were generated by limiting dilution of T-cell lines of donor 2 which were directed against peptide 62. Cells were stimulated with peptide pre-pulsed (10 µg/ml for 3 hours), irradiated (30Gy) allogeneic feeders in the presence of IL-2 (125 U/ml; Cetus). Cell growth in the 0.1 cell/well conditions was considered clonal, although formal clonality assessment was not performed. Cells were maintained in IL-2 and tested for suppression and cytolytic activity at the end of the stimulation cycle.

### Flow cytometry

T-cell lines were analyzed in detail by flowcytometry both directly from culture as well as after overnight restimulation with αCD3/28 beads (Dynal T-cell expander beads, Invitrogen). Antibodies used for staining included CD3-Pacific blue/ PE-Cy7, CD8-Am Cyan, CD25-APC-Cy7, CD56-PE-Cy5, CD94-PE, CD16-Pacific blue, TCRαβ-FITC, IFNγ-Alexa 700 (all BD Biosciences). Moreover, we used NKG2A-PE from Immunotech (Mijdrecht, The Netherlands), NKG2C-PE and NKG2D-PE (R&D systems, Abingdon, UK). In addition cells were stained for LAG-3 (17B4 kind gift of Dr. F. Triebel, Immutep S.A., Chatenay-Malabry, France) in combination with goat-anti-mouse-PE (Dako Cytomation, Heverlee, Belgium); CCL4-FITC (R&D systems), FoxP3-Pe-Cy5 (Ebioscience, San Diego, CA), Granzyme B-APC (CLB, Amsterdam, The Netherlands), rabbit anti-human granulysin (kind gift of Dr. A. Krensky, Stanford, CA) in combination with goat-anti-rabbit FITC (BD Biosciences). Membrane bound TGF-β1 was detected using the PE conjugated monoclonal TB21 (IQproducts, Groningen, The Netherlands). Intracellular staining was done after overnight incubation with Brefeldin A (3 µg/ml, Sigma) using Intrastain reagents (Dako Cytomation). Samples were acquired on a LSRII flowcytometer and analyzed using FACSDiva software (BD Biosciences). Scoring in Figure 3B was based on the percentage of cells that expressed the particular marker, markers expressed by 10% of the cells, or more, were considered positive.

### Cytotoxicity assays

Single HLA-E expressing cells lines (K562 cells) were used to test the capacity of the T-cell lines to lyse target cells that present the peptide in HLA-E (kind gift of Dr. E. Weiss, Ludwig-Maximilians-Universität, Munich, Germany) [59]. Lines were made by transfection of HLA-E (G variant = HLA-E*0103) in K562 cells, a leukemic line that does not express any HLA-class I molecules as previously described [59]. In order to obtain stable surface expression of HLA-E one of its natural ligands needs to be co-expressed; in this case this was achieved by cotransduction of the signal peptide of HLA-B7. HLA-E expression in HLA-E transfectants was inducible by culturing the cells at 26°C for at least one day. Subsequently predicted HLA-E binding Mtb peptides (10 µg/ml) were added for 16 hours at 26°C and HLA-E-peptide dependent expression was stabilized by a final incubation at 37°C for 2 hours. Transfectants were cultured in Iscove's modified Dulbecco's medium (Invitrogen, Breda, The Netherlands) supplemented with 10% FCS (Greiner Bio-One B.V., Alphen aan de Rijn, The Netherlands) and 200 µg/ml G418 (Invitrogen). Transfectants were always checked for HLA-E expression prior to experiments using FACS staining for HLA-class I (W6/32) and more specifically for HLA-E (3D12, kind gift of Dr. D. Geraghty, Fred Hutchinson Cancer Research Center, Seattle, WA).

Peptide loaded HLA-E transfectants were labeled with 1 µCi ^51^Cr before co-culture with different ratios of T-cell lines. After 4 to 5 hours of co-culture ^51^Cr release was measured and specific lysis calculated using the following formula: (sample lysis – medium)/(maximum lysis – medium) [60].

To demonstrate processing and recognition of naturally presented epitopes. T-cell lines were tested for recognition of BCG infected monocytes. Allogeneic PBMCs, completely mismatched for HLA-A, B & C (including supertypes) for all T-cell donors, and derived from 2 different PBMC donors, were plated for 7 days (150.000 cells/ well in 96 well plates) to obtain adherent monocytes. After washing cells were infected with live BCG (MOI 5, Montreal strain [32]) and labeled with 1 µCi ^51^Cr over night. After washing, cells were co-cultured for 5 hours with T-cell lines and assessment of ^51^Cr release. A control cytotoxic CD8^+^ T cell clone, reactive with the male HY antigen presented by HLA-A2 was included to control for aspecific lysis of infected monocytes (kindly provided by the laboratory of Prof E. Goulmy, department of Immunohematology and Blood Transfusion, LUMC, Leiden, previously described in [61]). HLA-A2 positive B-cells expressing the male HY antigen were used as natural targets for this CTL clone.

### Suppression assays

T-cell lines specific for predicted HLA-E binding peptides were tested for their capacity to inhibit proliferation of a well characterized Th1 responder clone (Rp15 1-1) [33]. T-cell lines were added in various doses to co-cultures containing 1×10^4^ Th1 cells, 5×10^4^ HLA-DR3 matched, irradiated PBMCs as APCs (20 Gy) and 0.5 µg/ml specific Mtb hsp65 p3–13 peptide. After 3 days, proliferation was measured by [^3^H] TdR incorporation.

To test if the reduced thymidine uptake by the responder T-cell clone (Rp15 1-1) was not the consequence of cell lysis we performed a CFSE labeling experiment, which included a control CD4^+^ T-cell clone (R2F10, HLA-DR2 restricted, specific for *M. leprae* hsp65 p418–427) as indicator for the input signal. Rp15 1-1 cells were labeled with a low dose CFSE (0.005 µM), whereas R2F10 was labeled with a high dose of CFSE (5 µM). Both CFSE labeled responder and control clones were co-cultured with the Mtb hsp65 p3–13 peptide, HLA-DR3 matched APCs and different concentrations of Tregs. After 16 hours (before divisions of the responder clone occur) cells were harvested, stained for CD3, CD4 and CD8 and analyzed by flowcytometry on a BD LSRII. The number of cells of both responder and control T-cell clones were compared: a ratio close to 1 indicates that the responder clone is not lysed by the Tregs, a ratio below 1 indicates lysis of the responder clone. Lysis of both responder and control T-cell clone can be excluded by comparison of conditions with and without Tregs.

To decipher the mechanism of suppression mediated by the HLA-E restricted Tregs, we performed inhibition experiments with the Latency Associated Peptide (LAP) [38]. LAP binds to TGFβ1 and thereby limits its activity. In these experiments, Tregs were titrated onto the Rp15 1-1 responder T-cell clone, in the presence of peptide (0.5 µg/ml), APCs and different concentrations of LAP (R&D systems); [^3^H] TdR incorporation was assessed after 3 days of co-culture.
